# How many laboratories are necessary? Analytical assessment of precision and reproducibility in disinfectant efficacy testing for quantifying the impact of laboratory number on result reliability

**DOI:** 10.3205/dgkh000655

**Published:** 2026-06-05

**Authors:** Kira-Marie Roesch, Marvin Rausch, Felix Droop, Martin Exner, Stefanie Gemein, Carola Ilschner, Axel Kramer, Thomas Selhorst, Miranda Suchomel, Nico T. Mutters, Jürgen Gebel

**Affiliations:** 1 University Bonn, University hospital Bonn, Institute for Hygiene and Public Health, Bonn, Germany; 2 VAH – Association for Applied Hygiene, Germany; 3 University of Bonn, Germany; 4 Referenzinstitut für Bioanalytik, Bonn, Germany; 5 Institute of Hygiene and Environmental Medicine, University Medicine Greifswald, Germany; 6 Federal Institute for Risk Assessment, Berlin, Germany; 7 Institute for Hygiene and Applied Immunology, Medical University of Vienna, Vienna, Austria

**Keywords:** disinfectant efficacy testing, precision and reproducibility, analytical modelling, ring trials, interlaboratory variability, intralaboratory variation, confirmation probability, quality assurance

## Abstract

**Background:**

Reliable assessment of the efficacy of chemical disinfection procedures requires robust experimental precision and reproducibility. In order to obtain a certificate from the Association for Applied Hygiene (VAH) proving the efficacy of a disinfectant procedure, test results from two independent accredited laboratories must be submitted for evaluation. In a previous study, Monte-Carlo simulations based on VAH ring trial data demonstrated that this two-laboratory stipulation significantly reduces the probability of false-positive efficacy statements. The present study extends that work by developing an analytical solution to quantify the influence of intra- and interlaboratory variability on efficacy assessment outcomes.

**Methods:**

The analysis is based on data from four VAH ring trials covering bactericidal, yeasticidal, and mycobactericidal activities as tested according to EN 13727, EN 13624, EN 17387 and EN 14563. Analytical probability models were derived to calculate the likelihood that one, two, three, or four laboratories independently classify a disinfectant as effective under defined test conditions. The results were compared across different efficacy ranges and interpreted in relation to the existing two-laboratory validation framework.

**Results:**

The analytical solution confirms that including a second laboratory significantly enhances the robustness of efficacy classification by reducing the probability of false-positive outcomes in the intermediate efficacy range by up to 90%. Beyond two laboratories, the benefit is observed to drop sharply: Inclusion of a third or fourth laboratory further lowers the probability of consistent positive classifications, but the marginal gain in reliability is small relative to the associated increase in cost, effort, and organizational complexity. The model thus reveals an asymptotic relationship between the number of laboratories and classification certainty.

**Conclusion:**

The analytical findings corroborate the empirical findings from VAH ring trials, demonstrating that the two-laboratory stipulation represents an optimal balance between statistical reliability and practical feasibility. Expanding beyond two laboratories provides minimal additional certainty and may increase disagreement rates due to compounding intralaboratory variation. Future standardization efforts should therefore prioritize the reduction of intralaboratory variability rather than expanding interlaboratory replication.

## Zusammenfassung

**Hintergrund:**

Eine zuverlässige Bewertung der Wirksamkeit chemischer Desinfektionsverfahren erfordert eine hohe experimentelle Präzision und Reproduzierbarkeit. Der Verbund für Angewandte Hygiene (VAH) fordert als Voraussetzung für eine Produktzertifizierung Testergebnisse von zwei unabhängigen akkreditierten Laboren. In einer früheren Studie haben Monte-Carlo Simulationen auf der Grundlage von VAH-Ringversuchsdaten gezeigt, dass diese Forderung zur Prüfung durch zwei Labore die Wahrscheinlichkeit falsch-positiver Wirksamkeitsaussagen erheblich verringert. Die vorliegende Studie erweitert diese Arbeit durch die Entwicklung einer analytischen Lösung zur Quantifizierung des Einflusses der Variabilität innerhalb und zwischen Laboren auf die Ergebnisse der Wirksamkeitsbewertung.

**Methoden:**

Die Analyse basiert auf Daten aus vier VAH-Ringversuchen zur Bestimmung der bakteriziden, levuroziden und mykobakteriziden Wirkung gemäß den europäischen Testmethoden EN 13727, EN 13624, EN 17387 und EN 14563. Es wurden analytische Wahrscheinlichkeitsmodelle abgeleitet, um die Wahrscheinlichkeit zu berechnen, dass ein, zwei, drei oder vier Laboratorien ein Desinfektionsmittel unter definierten Testbedingungen unabhängig voneinander als wirksam einstufen. Die Ergebnisse wurden über verschiedene Wirksamkeitsbereiche hinweg verglichen und im Zusammenhang mit dem bestehenden Validierungsrahmen für zwei Laboratorien interpretiert.

**Ergebnisse:**

Die analytische Lösung bestätigt, dass die Einbeziehung eines zweiten Labors die Robustheit der Wirksamkeitsklassifizierung deutlich verbessert, indem sie die Wahrscheinlichkeit falsch-positiver Ergebnisse im intermediären Wirksamkeitsbereich um bis zu 90% reduziert. Bei mehr als zwei Laboren nimmt der Nutzen jedoch stark ab: Die Einbeziehung eines dritten oder vierten Labors senkt die Wahrscheinlichkeit konsistenter positiver Klassifizierungen weiter, aber der marginale Gewinn an Zuverlässigkeit ist im Verhältnis zu den damit verbundenen höheren Kosten, höherem Aufwand und größerer organisatorischer Komplexität gering. Das Modell zeigt somit eine asymptotische Beziehung zwischen der Anzahl der Labore und der Klassifizierungssicherheit.

**Schlussfolgerung:**

Die analytischen Ergebnisse untermauern die Erkenntnisse aus VAH-Ringversuchen und zeigen, dass die Zwei-Labor Entscheidung ein optimal ausgewogenes Verhältnis von statistischer Zuverlässigkeit und notwendigem Aufwand darstellt. Die Erweiterung über zwei Labore hinaus bietet nur minimale zusätzliche Sicherheit und kann aufgrund der sich verstärkenden intralaboratorischen Variation zu einer Erhöhung der Diskrepanzen führen. Zukünftige Standardisierungsbemühungen sollten daher der Verringerung der Variabilität innerhalb der Labore Vorrang vor der Ausweitung der Replikation zwischen den Labors einräumen.

## Introduction

Reliable assessment of the efficacy of chemical disinfection procedures requires a high level of precision and reproducibility, as even minor methodological or biological variations can substantially influence test outcomes and thus the efficacy of the disinfection measure. The current approach adopted by the Association for Applied Hygiene (VAH), which mandates test results from two independent accredited laboratories [1], has proven to be an important quality assurance measure. The previous study, “Requirements for the precision and reproducibility in the efficacy testing of chemical disinfection procedures” [2], demonstrated through Monte-Carlo simulations based on VAH ring trial data that the inclusion of a second laboratory substantially reduces the probability of misclassification and results in more conservative and reproducible efficacy evaluations.

While the prior publication focused on simulation-based estimations of uncertainty, the present study emphasizes an analytical statistical approach to further elucidate the mechanisms underlying inter- and intralaboratory variability. By deriving closed-form probability expressions, the analysis describes how variance structures within and between laboratories determines the likelihood that one, two, three, or more laboratories independently classify a procedure as effective. This approach allows the confirmation probability of efficacy assessments to be expressed explicitly as a function of laboratory number and underlying variability.

A central focus of the present study is the behaviour of efficacy classifications within decision-critical result ranges, that is, concentration–time conditions close to the regulatory efficacy threshold. In these ranges, laboratory-related variability exerts a disproportionate influence on classification outcomes, such that additional laboratories may potentially have a significant impact on the probability of achieving concordant efficacy decisions. The analytical model therefore permits a systematic evaluation of how expanding the test design from one to multiple laboratories affects classification certainty and agreement.

This issue is not only of theoretical but also of practical as well as of regulatory importance. On the one hand, including more laboratories may enhance the validity of efficacy assessments within decision-critical result ranges. On the other hand, should an increasing number of laboratories be consecutively involved in the decision-making process, it can be assumed that the disinfection procedure in question will ultimately be rejected, on the condition that a minimum of one of the total numbers of laboratories involved classifies the disinfectant procedure as ineffective. An analytical examination of these relationships allows for a deeper understanding of the balance between methodological robustness, statistical significance, and economic feasibility. 

The present work therefore builds directly upon the VAH dataset and prior findings from ring trials to provide an analytically derived model for precision and reproducibility in disinfectant efficacy testing. The overall goal is to determine whether increasing the number of test laboratories yields a measurable improvement in the reliability of efficacy classification or whether, beyond a certain threshold, the incremental benefit no longer justifies the additional complexity, cost, and resource requirements. These results are expected to inform future standardization efforts and contribute to the evidence-based refinement of regulatory frameworks within the European and international context.

## Method

### Data basis and study context

The present analysis builds upon the same dataset used in our previous study [2], which evaluated interlaboratory variability in disinfectant efficacy testing using data from four VAH ring trials [3]. These ring trials, organized by the VAH, were conducted over several years to assess the precision and reproducibility of standardized quantitative test methods for chemical disinfectants and antiseptics in accordance with European standards. The goal of the VAH ring trial program is to ensure method reliability, evaluate laboratory performance under harmonized test conditions, and provide empirical data to support quality assurance and accreditation within microbiological efficacy testing.

### VAH ring trials and test design

The analytical evaluation presented here uses data from four VAH ring trials, each representing distinct microorganism categories and methodological conditions (Table 1 (Tab. 1)) [3], [4], [5], [6], [7].

All participating laboratories received identical test products from a single production batch, provided and distributed by the VAH. Each laboratory performed quantitative efficacy tests according to the selected European standard under controlled and standardized conditions. Reduction factors (R; lg-reductions) were determined for each test product and condition. The datasets thus capture both intra- and interlaboratory sources of variation under simulated-use testing conditions representative of the VAH certification framework.

### Analytical solution

Variability arises both intralaboratory and interlaboratory, raising the question of whether efficacy assessments based on a single laboratory are sufficiently reliable. To increase the robustness of the evaluation process, it is advisable to involve multiple independent laboratories. Concordant results across laboratories reduce the risk of misclassification.

In this context, the study compares two decision rules:

- one-laboratory rule: This regulatory approach designates a single laboratory—selected at random—as the sole authority for the efficacy assessment
- two-laboratory rule: Under this rule, a substance is classified as effective only if both independent laboratories unanimously report it as effective
- X-laboratory rule: Under this rule, a substance is classified as effective only if X independent laboratories unanimously report it as effective

The proportion of laboratories of a ring trial is *N*. The laboratories with stated the product effective (R≥5 lg for bactericidal efficacy and R≥4 lg for yeasticidal efficacy) and not effective (R<5 lg accordingly R<4 lg) was then determined. 

Under the one-laboratory rule, an effective assessment corresponds to the event that the first laboratory drawn belongs to the laboratories which classified the disinfection procedure as effective (*N_eff_*). The corresponding confirmation probability *p1* is


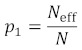


Under the two-laboratory rule, an effective assessment corresponds to the event that, after one laboratory has been drawn and classified the procedure as effective, a second laboratory drawn without replacement from the remaining pool also classifies the procedure as effective. The corresponding confirmation probability therefore reflects this sequential sampling process. Owing this procedure, the corresponding confirmation probability is


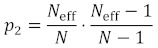


Where


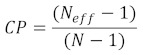


defines the confirmation probability (CP) of the second laboratory.

Accordingly, 1–CP quantifies the probability that an effective classification under the one-laboratory rule is not confirmed when a second laboratory is required. 

For a specific number of laboratories *k*>2 the confirmation probability of an effective final classification *p_k_* is: 


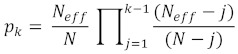


## Results

### Intralaboratory instead of interlaboratory variability

In a previous paper, we showed that statistical analyses using one-way analysis of variance (ANOVA) demonstrated that the laboratory itself exerts a statistically significant influence on the measured reduction values across all four ring trials (p<0.001 for all test types and efficacy ranges) [2]. This finding confirmed substantial interlaboratory variation, reflecting differences in execution, equipment, environmental conditions, and operator-specific factors despite adherence to standardized protocols. On this basis, the present study no longer focuses on variation between laboratories (interlaboratory) but rather on variability within a laboratory (intralaboratory), the variation that occurs in repeated measurements within a single laboratory. Using an analytical statistical framework, this work aims to derive a closed-form solution that describes how such intralaboratory variability affects the precision and reliability of efficacy classification when expanding from one to multiple laboratory datasets.

The analytical model used in this study therefore provides a mathematical complement to the simulation-based findings of the prior publication, quantifying the trade-off between increasing the number of laboratories involved and the resulting statistical certainty of the efficacy decision.

### Analytical solution of the ineffective range

For the ineffective range of each ring trial, the proportion of laboratories in VAH ring trials with a decision of ineffective is 100%. No simulation was carried out here, as the decision ineffective was unanimous (Table 2 (Tab. 2)). 

### Analytical solution of the intermediate effective range

**Figure 1:**
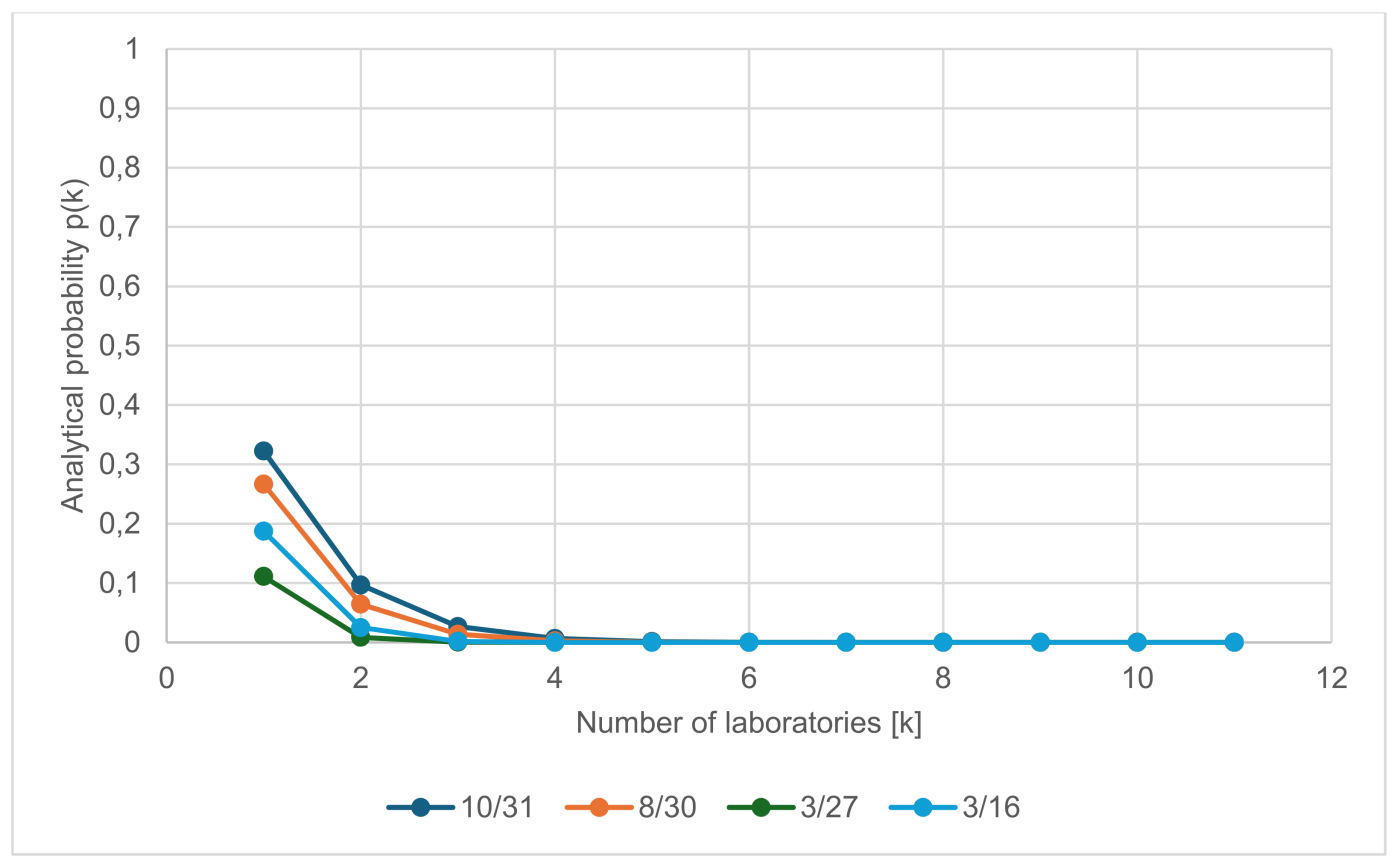
Analytical probability of laboratories that show effective results within the intermediate effective range of each VAH ring trial in the scheme Neff/N [dark blue=R2023-01, orange=R2020-01, green=R2022-01, light blue=R2019-02].

For the intermediate effective range of each ring trial, the proportion of laboratories in VAH ring trials with a decision of effective is shown in Table 2 (Tab. 2). The results of the analytical solution for one, two, three and four laboratories can be found in Table 3 (Tab. 3) and Figure 1 (Fig. 1) calculated according to Equation 1–4.

### Probability for classification as effective in the different ring trials

- R2023-01: The analytical solution shows that the probability that a single laboratory classifies a procedure as effective is 32.3%. The probability that two independently selected laboratories both classify the same procedure as effective is 9.7%, and the probability that three laboratories do so is 2.7% and with four laboratories 0.7% (see Table 3 (Tab. 3) and Figure 1 (Fig. 1)). The probability that a second, independently selected laboratory also classifies a procedure as effective, given that the first laboratory has already classified it as effective is 30.0%.
- R2020-01: The analytical solution in comparison describes the probability of effective results for one laboratory of 26.7%, reducing to 6.4% with a second laboratory and further reducing to 1.3% and 0.2% with inclusion of a third or fourth laboratory (see Table 3 (Tab. 3) and Figure 1 (Fig. 1)). The probability that a second, independently selected laboratory also classifies a procedure as effective, given that the first laboratory has already classified it as effective is 24.1%.
- R2022-01: The analytical solution in comparison describes the probability of effective results for one laboratory of 11.1%, reducing to 0.9% with a second laboratory and further reducing to 0.0% with inclusion of a third or fourth laboratory (see Table 3 (Tab. 3) and Figure 1 (Fig. 1)). The probability that a second, independently selected laboratory also classifies a procedure as effective, given that the first laboratory has already classified it as effective is 7.8%.
- R2019-02: The analytical solution in comparison describes the probability of effective results for one laboratory of 18.8%, reducing to 2.5% with a second laboratory and further reducing to 0.2% or 0.0% with inclusion of a third or fourth laboratory (see Table 3 (Tab. 3) and Figure 1 (Fig. 1)). The probability that a second, independently selected laboratory also classifies a procedure as effective, given that the first laboratory has already classified it as effective is 13.3%.

### Analytical solution of the effective range

**Figure 2:**
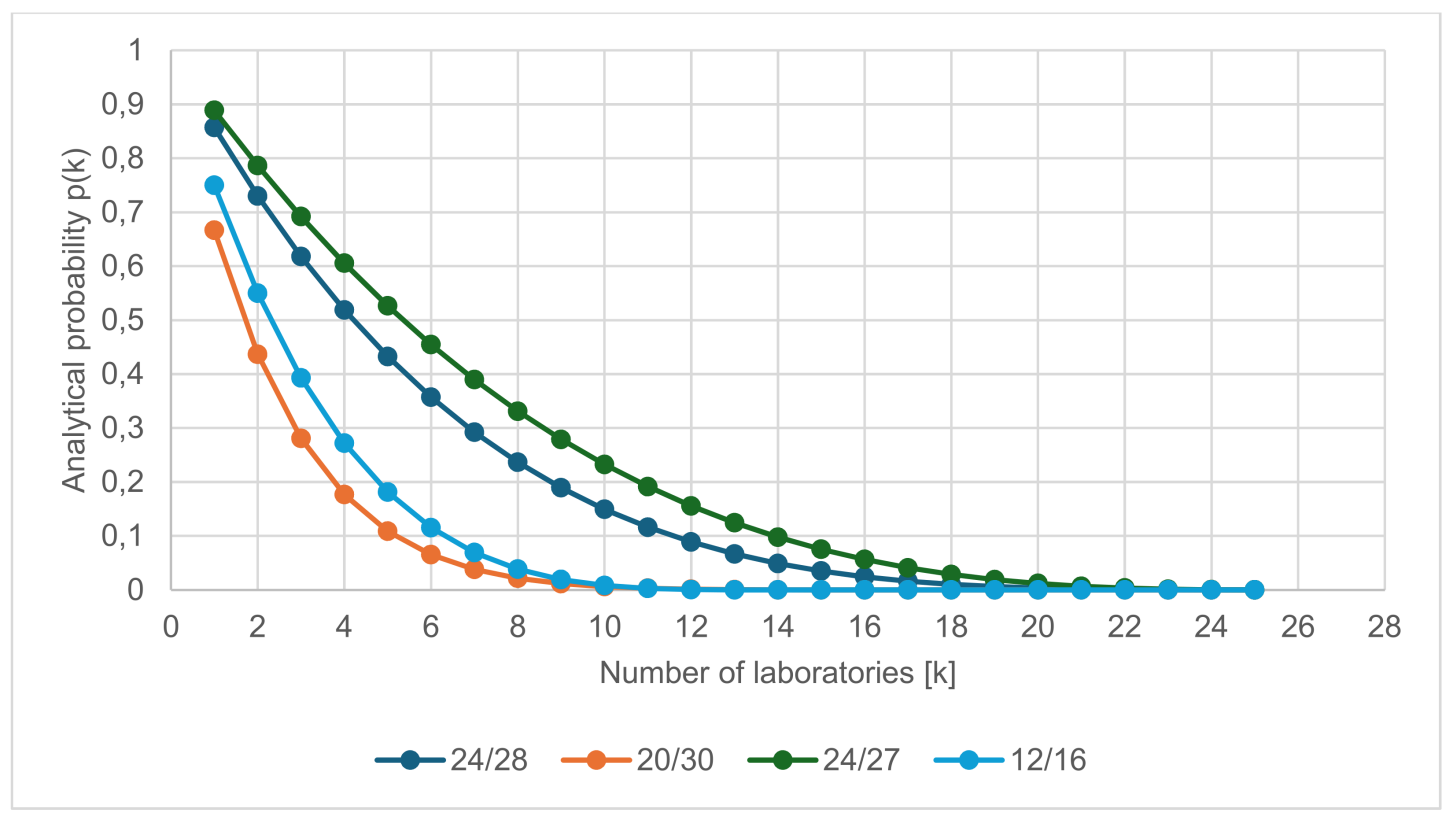
Analytical probability of laboratories that show effective results within the effective range of each VAH ring trial in the scheme Neff/N [dark blue=R2023-01, orange=R2020-01, green=R2022-01, light blue=R2019-02]

For the effective range of each ring trial, the proportion of laboratories in VAH ring trials with a decision of ineffective is shown in Table 2 (Tab. 2). The results of the analytical solution for one, two, three or four laboratories can be found in Table 4 (Tab. 4) and Figure 2 (Fig. 2) calculated according to Formula 1–4.

- R2023-01: The analytical solution in comparison describes the probability of effective results for one laboratory of 85.7%, reducing to 73.0% with a second laboratory and further reducing to 61.9% or 51.9% with inclusion of a third or fourth laboratory (see Table 4 (Tab. 4) and Figure 2 (Fig. 2)). The probability that a second, independently selected laboratory also classifies a procedure as effective, given that the first laboratory has already classified it as effective is 85.2%.
- R2020-01: The analytical solution in comparison describes the probability of effective results for one laboratory of 66.7%, reducing to 43.7% with a second laboratory and further reducing to 28.1% or 17.7% with inclusion of a third or fourth laboratory (see Table 4 (Tab. 4) and Figure 2 (Fig. 2)). The probability that a second, independently selected laboratory also classifies a procedure as effective, given that the first laboratory has already classified it as effective is 65.5%.
- R2022-01: The analytical solution in comparison describes the probability of effective results for one laboratory of 88.9%, reducing to 78.6% with a second laboratory and further reducing to 69.2% or 60.5% with inclusion of a third or fourth laboratory (see Table 4 (Tab. 4) and Figure 2 (Fig. 2)). The probability that a second, independently selected laboratory also classifies a procedure as effective, given that the first laboratory has already classified it as effective is 88.5%.
- R2019-02: The analytical solution in comparison describes the probability of effective results for one laboratory of 75.0%, reducing to 55.0% with a second laboratory and further reducing to 39.3% or 27.2% with inclusion of a third or fourth laboratory (see Table 4 (Tab. 4) and Figure 2 (Fig. 2)). The probability that a second, independently selected laboratory also classifies a procedure as effective, given that the first laboratory has already classified it as effective is 73.3%.

### Analytical solution of a theoretical data set

**Figure 3:**
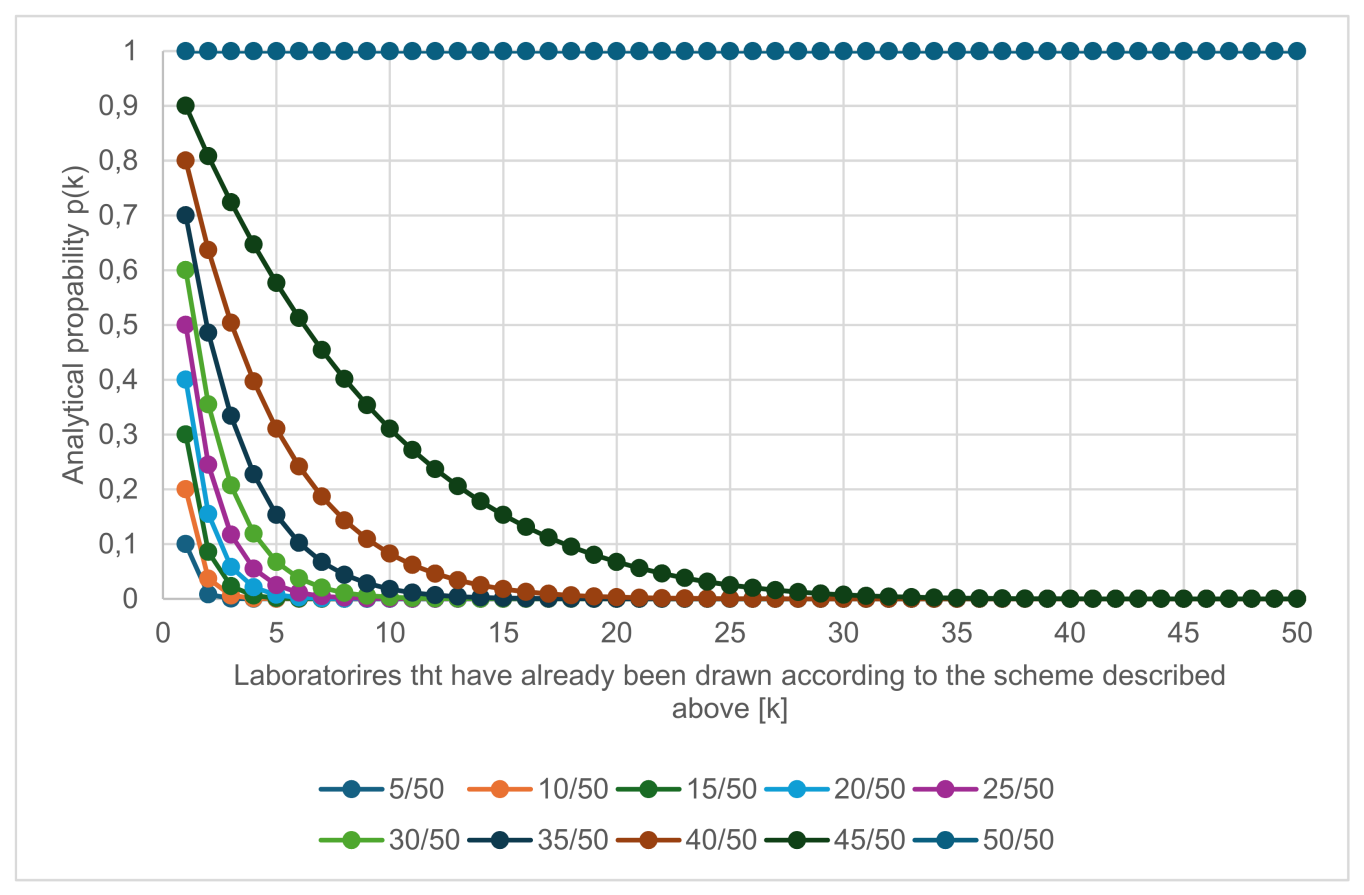
Analytical probability of laboratories that show effective results within a theoretical data set in steps of 5 effective laboratories till 50 laboratories in the scheme N_eff_/N.

To see how the probability of equal outcomes changes depending on what the outcome of a dataset looks like, Figure 3 (Fig. 3) shows this graphically for illustration purposes.

## Discussion

The analytical evaluation presented here builds upon the findings of the VAH ring trials and the simulation-based results of the previous publication [2]. While the prior analysis demonstrated by means of Monte-Carlo simulation that the inclusion of a second independent laboratory significantly reduces the probability of overly optimistic efficacy classifications, the present study provides a closed-form analytical solution that allows the same relationships to be expressed and interpreted mathematically.

The VAH ring trial program, which systematically investigates precision and reproducibility across multiple laboratories, has shown that variability in disinfection efficacy testing is not solely random but influenced by identifiable factors intrinsic to each laboratory [8], [9]. In the earlier analysis, one-way ANOVA revealed that the laboratory itself significantly affects the outcome (*p*<0.001 in all ring trials), confirming the presence of substantial interlaboratory variation even under standardized testing conditions. Building on this foundation, the current analytical approach identifies intralaboratory variability as a key determinant of the overall uncertainty in efficacy classification and quantifies how this uncertainty propagates when the results from additional laboratories are considered in the decision process regarding efficacy.

Across all four ring trials, the analytical results confirm that including a second independent laboratory significantly increases the robustness of efficacy classification by reducing the probability of a product being incorrectly assessed as effective in the intermediate range (Table 3 (Tab. 3)). For instance, in trial R2023-01, the probability that a single laboratory classified a product as effective was 32.3%, whereas the probability that two laboratories independently agreed on such a classification dropped to 9.7%. These results corroborates the interpretation that multi-laboratory evaluation serves as an effective corrective mechanism for random or systematic laboratory-level bias.

The analytical results for the effective range reveal a similar but less pronounced trend. The likelihood that two laboratories independently confirm efficacy decreases moderately compared with single-laboratory assessment, reflecting the higher inherent stability of test results in clearly effective conditions (Table 4 (Tab. 4)). For example, in R2023-01 the probability of an effective classification decreased from 85.7% for one laboratory to 73.0% for two laboratories. The CP that a second laboratory confirms the first laboratory’s positive result was 85.2%, indicating that even under optimal conditions, full agreement is not guaranteed. Similarly, CP values of 88.5% (R2022-01) and 73.3% (R2019-02) demonstrate that residual experimental uncertainty persists across all efficacy levels.

When extending the analytical model to include three and four laboratories, the diminishing marginal effect of additional testing becomes evident. The probability that three laboratories independently agree on an efficacy classification decreases sharply across all trials. While the CP correspondingly increases, suggesting that the result becomes more conservative from a statistical point of view, the rate of matching classifications decreases. In the intermediate effective range, the inclusion of a third laboratory reduced the probability of an efficacy classification to between 0.0% (R2022-01) and 2.7% (R2023-01). The probability that three laboratories independently confirm a positive result is therefore extremely low, underscoring how additional replication exponentially decreases the likelihood of unanimous “effective” assessments in borderline efficacy conditions. The probability if four laboratories need to be effective is less than 1 of each of the ring trials in the intermediate range (see Table 3 (Tab. 3)). 

For the effective range, the same trend persists but to a lesser extent. The probability of efficacy classification for three laboratories ranged from 28.1% (R2020–01) to 69.2% (R2022-01), while projections for four laboratories indicate further moderate reductions to 17.7%–60.5%, depending on the microbial spectrum and test type. The analytical solution thus highlights a law of diminishing returns: beyond two laboratories, the additional gain in reliability is progressively outweighed by an increased probability of non-oncordant outcomes caused by random intralaboratory fluctuations. This relationship reflects the mathematical property of multiplicative probability reduction, where independent errors or variations increase with the number of laboratories.

From a methodological and regulatory point of view, these results suggest that the requirement of two independent accredited laboratories, as specified by VAH and the Commission for Infection Prevention and Hygiene in Healthcare and Nursing (KRINKO) [10] and similar approaches adopted by the German Veterinary Medical Society (DVG) [11], the Organization for Economic Co-operation and Development (OECD) [12], the Austrian Society for Hygiene, Microbiology and Preventive Medicine (ÖGHMP) [13], the U.S. Environmental Protection Agency (EPA) and the U.S. Food and Drug Administration (FDA) [14], represents an optimal balance between analytical robustness and practical feasibility. This aspect is particularly relevant in the context of the European Biocidal Products Regulation (BPR), which promotes active substance minimization and thus leads to products being positioned close to the efficacy threshold. The present data demonstrate that precisely in these decision-critical ranges, variability between laboratories is highest, increasing the risk of inconsistent classifications when relying on a single laboratory. The requirement for two independent laboratories therefore represents a scientifically justified and practically appropriate standard [15]. While expanding to three or four laboratories theoretically yields a more conservative decision threshold, the corresponding increase in cost, time, and organizational complexity is unlikely to be justified by the marginal improvement in statistical confidence. Moreover, excessive replication may paradoxically amplify the perception of uncertainty by reducing the frequency of unanimous positive results, despite no change in the underlying efficacy of the product. These effects are confirmed by the theoretical data set, presented in Figure 3 (Fig. 3), were scenarios with different numbers of effective results are calculated from a total of 50 potential data sets or laboratories. The fewer effective data sets there are, the more difficult it becomes for two or more identical results to be generated independently of each other.

In a broader context, the analytical model underscores the necessity of distinguishing between variability that can be reduced through methodological standardization (e.g., consistent inoculum preparation, media quality, and procedural training) and variability that is inherent to biological test systems and thus cannot be fully eliminated. While ring trials remain an indispensable tool for quality assurance and standardization, the analytical framework introduced here offers a complementary instrument for predicting and managing the uncertainty inherent in multi-laboratory efficacy assessments.

Ultimately, this study demonstrates that the analytical solution confirms the two-laboratory rule as an empirically and theoretically justified standard for disinfectant efficacy evaluation. More than two laboratories, while potentially beneficial in special cases, appears to yield progressively smaller improvements in classification reliability. 

In this respect, the present study complements prior simulation-based evidence with an exact analytical foundation, offering a rigorous mathematical justification for the existing VAH approach and providing basis for refining future guidelines on disinfection efficacy testing.

## Conclusions

The analytical results presented here provide a quantitative foundation for understanding the relationship between laboratory number and the reliability of disinfectant efficacy classification. Building on extensive VAH ring trial data, the analytical model demonstrates that while the inclusion of a second independent laboratory substantially improves classification robustness and minimizes the likelihood of false-positive efficacy assessments, further expansion to three or four laboratories yields only marginal additional benefit.

Beyond two laboratories, the gain in statistical certainty approaches an asymptotic limit, while the probability of discordant outcomes increases disproportionately due to compounding intralaboratory variation. These findings confirm that the two-laboratory stipulation, as currently required by the VAH and recommended by KRINKO [1], [10] and other organisations, represents an empirically and analytically optimal standard for ensuring reproducibility without an unnecessary burden of testing costs and complexity.

From a regulatory perspective, this analytical framework underscores the importance of directing future quality assurance endeavours towards reducing intralaboratory variation. This can be archived through standardization of test conditions, the provision of operator training, and the harmonization of test procedures, rather than expanding the number independent laboratories and expert opinions. Strengthening reproducibility within laboratories is deemed to be more efficacious with regard to enhancing the precision of efficacy testing than merely increasing interlaboratory replication beyond two laboratories.

In summary, the study provides further theoretical and empirical justification, in line with existing literature, for maintaining the two-laboratory rule as the cornerstone of reliable disinfectant efficacy assessment. The analytical solution offers a mathematically sound complement to simulation–based evidence and supports evidence-driven standardization in accordance with European and international disinfection testing frameworks.

## Notes

### Authors’ ORCIDs:

- Roesch K: https://orcid.org/0009–0000–3494–9191
- Rausch M: https://orcid.org/0000–0003–1562–4337
- Exner M: https://orcid.org/0000–0002–6383–7866
- Gemein S: https://orcid.org/0009–0006–7329–9404
- Ilschner C: https://orcid.org/0009–0006–4083–7405
- Kramer A: https://orcid.org/0000–0003–4193–2149
- Selhorst T: https://orcid.org/0000–0003–0969–1533
- Suchomel M: https://orcid.org/0000–0001–8758–9652
- Mutters NT: https://orcid.org/0000–0002–0156–9595
- Gebel J: https://orcid.org/0000–0001–9328–3174

### Funding 

This work was funded by the Association for Applied Hygiene (VAH), Germany. 

### Acknowledgments 

The authors are grateful for the input provided by all laboratories which participated in the VAH ring trials. 

### Competing interests

The authors declare that they have no competing interests.

### Authors’ contributions

The authors Roesch K and Rausch M contributed equally.

## Figures and Tables

**Table 1:**
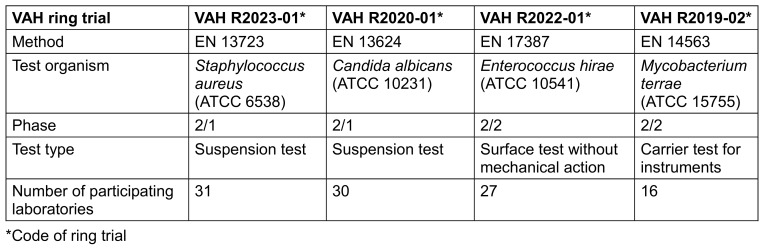
Test parameter for each VAH ring trial with number of participating laboratories.

**Table 2:**
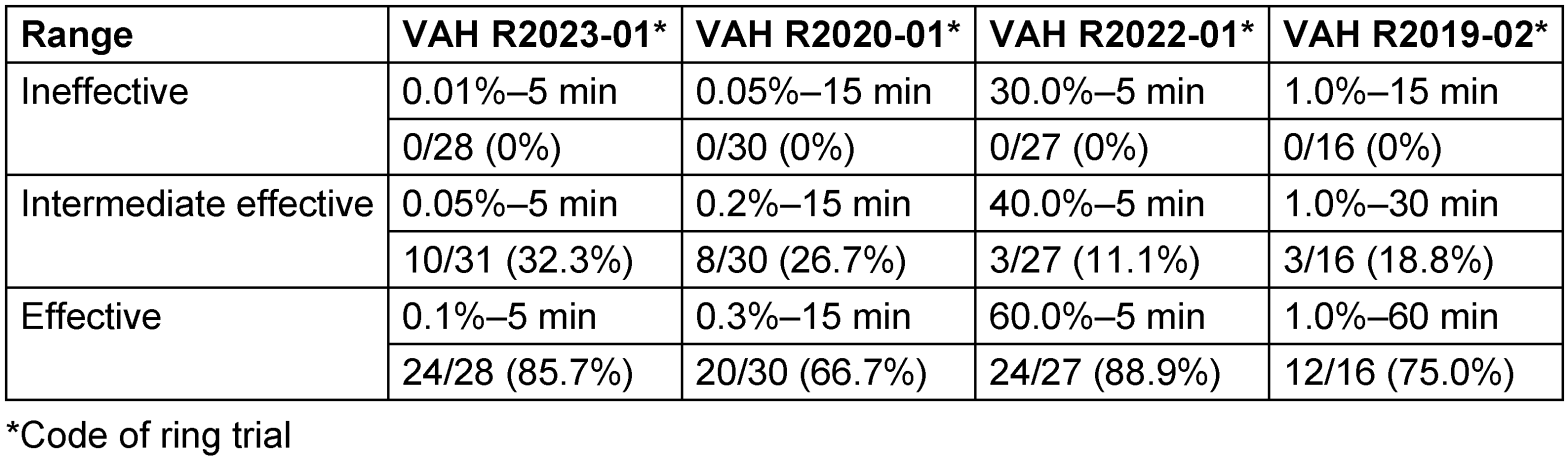
Number of laboratories in the different VAH ring trials and ranges which classified the tested product as effective.

**Table 3:**
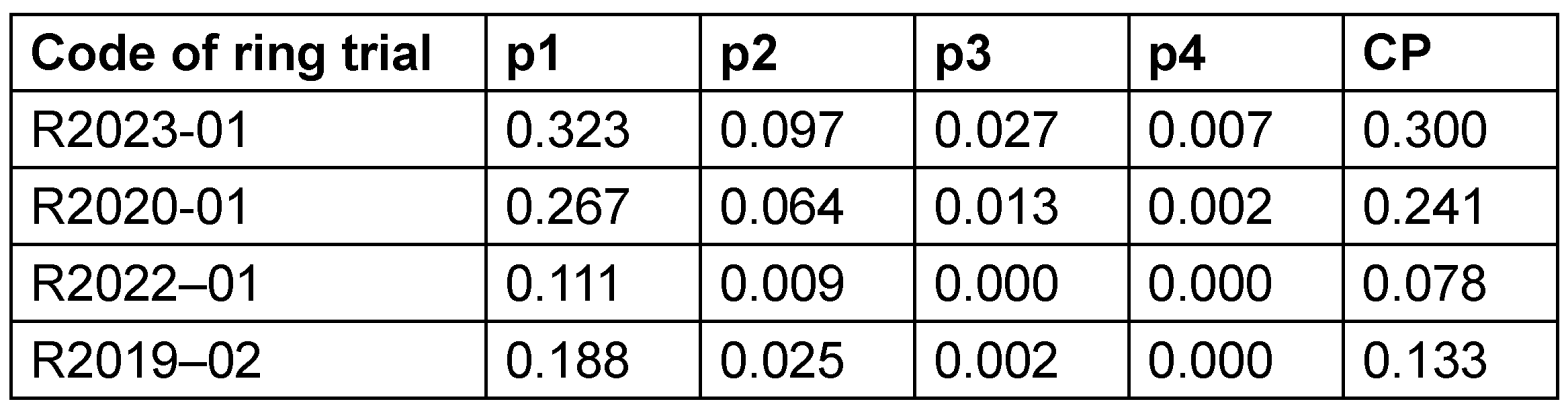
Results of the analytical solution for the intermediate effective range of each VAH ring trial [p=probability, CP=confirmation probability of 1 to 2].

**Table 4:**
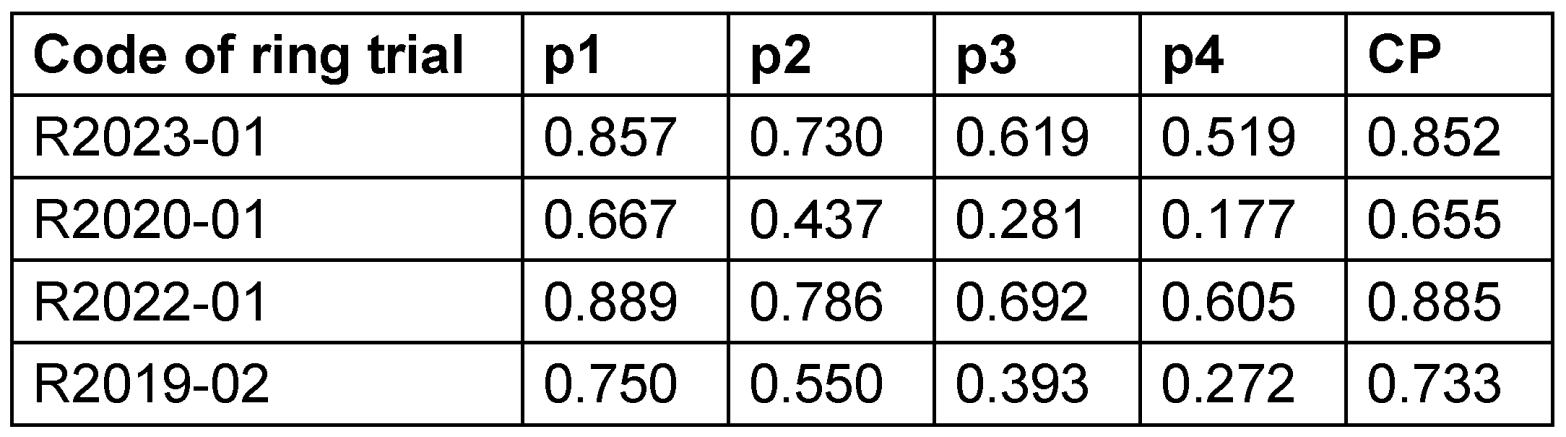
Results of the analytical solution for the effective range of each VAH ring trial. [p=probability, CP=confirmation probability from 1 to 2].
